# Pathophysiology of Greedy Colon and Diabetes: Role of Atropine in worsening of Diabetes

**DOI:** 10.5005/jp-journals-10018-1096

**Published:** 2014-01-22

**Authors:** Rohit Gundamaraju, Ravichandra Vemuri

**Affiliations:** 1 Department of Physiology, Faculty of Medicine, University of Malaya, Kuala Lumpur, Malaysia; 2 Department of Physiology, Faculty of Medicine, University of Malaya, Kuala Lumpur, Malaysia

**Keywords:** Diabetes, Greedy colon, Constipation, Atropine, Public health.

## Abstract

Greedy colon which is a synonym of constipation is a serious condition in the human body which may lead to complications, like damage of the rectal tissue, cellular dehydration and colorectal cancer. Diabetes mellitus, although a systemic disease with diverse clinical symptoms, is also related with cellular dehydration. Understanding the pathophysiological aspects of diabetes mellitus and greedy colon may shed light in the management of either of these conditions. The main purpose of this article is to demonstrate an association of tissue dehydration during diabetes mellitus and constipation. The adverse side effects of atropine will be discussed due to its M3 blockage effect and reduction in peristalsis keeping in mind the importance of these facts in the context of public health importance, especially in geriatric health.

**How to cite this article:** Gundamaraju R, Vemuri R. Pathophysiology of Greedy Colon and Diabetes: Role of Atropine in worsening of Diabetes. Euroasian J Hepato-Gastroenterol 2014;4(1):51-54.

## INTRODUCTION

Diabetes mellitus (DM) represents a group of metabolic disease in which a person express high blood sugar, either because the pancreas does not produce enough insulin or because the cells do not respond properly to the insulin.^1^ Diabetes mellitus possesses considerable health-related threat worldwide.^2^ Diabetes mellitus is one of the most common endocrine disorders which has caused significant morbidity and mortality due to microvascular (retinopathy, neuropathy and nephropathy) and macrovascular (heart attack, stroke and peripheral vascular diseases) complications.^3^ All forms of DM increase the risk of long-term complications. These typically develop after many years (10-20), but may be the first symptom in those who have otherwise not received a diagnosis before. The major long-term complications are related to damage to blood vessels. Diabetes mellitus doubles the risk of cardiovascular disease.^4^ The main ‘macrovascular’ diseases (related to atherosclerosis of larger arteries) are ischemic heart disease (angina and myocardial infarction), stroke and peripheral vascular disease. Diabetes also damages the capillaries (causes microangiopathy).^5^ Diabetic retinopathy, which affects blood vessel formation in the retina of the eye, can lead to visual symptoms, reduce vision and potentially blindness. Diabetic nephropathy, the impact of diabetes on the kidneys, can lead to scarring changes in the kidney tissue, loss of small or progressively larger amounts of protein in the urine, and eventually chronic kidney disease leading to damage and even failure of the organ requiring dialysis. Diabetic neuropathy is the affect of diabetes on the nervous system, most commonly causing numbness, tingling and pain in the feet and also increasing the risk of skin damage due to altered sensation. Together with vascular disease in the legs, neuropathy contributes to the risk of diabetes-related foot problems (such as diabetic foot ulcers) that can be difficult to treat and occasionally require amputation.

### Greedy Colon

In some persons, the fecal residues are greatly reduced in amount. This occurs in condition called as greedy colon.^6^ The normal and healthy form and consistency of the stool is that of soft mush. The so-called well-formed stool is the result of constipation. When the food residues of a meal are able to exit within 12 to 16 hours, they do have normal consistency. It is only when the materials remain in the lower colon for 24 or 48 hours, they become sufficiently dried. When the colon contents are retained so long, a large part of water is absorbed and the stool appears in the form of balls. This is the erroneous condition in the greedy colon.^7^

**Fig. 1:**
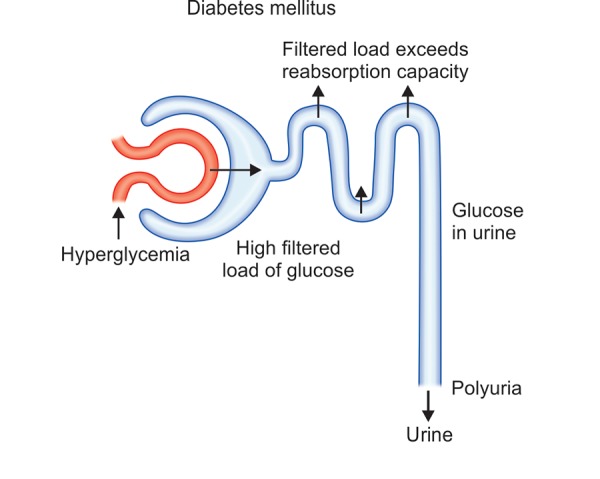
Condition of polyuria in diabetic patients

### Association of Diabetes and Constipation

Constipation is a known condition in individuals which occurs when there is an obstruction in the bowel movement. One condition of constipation in diabetes is the fact that various nerves regulate the duration of residual waste in the intestines. If these nerves have been damaged by high blood sugar levels, food and waste products may move through the intestines too slowly, causing constipation.

A recent systematic review showed that impairment caused by constipation as measured by Health-related Quality of Life scores predominates in the mental health domains and is comparable to that caused by serious chronic conditions, such as osteoarthritis and diabetes.^7^

### Mechanism involved in the Correlation of Greedy colon and diabetes

Diabetes is the condition associated with frequent urination polyuria, polydipsia which leads to excessive thirst.^8^ Under normal circumstances, 100% of the glucose that is filtered is reabsorbed. Glucose reabsorption involves transport proteins that require specific binding. In a diabetic patient that has hyperglycemia, the filtered load of glucose (amount of glucose filtered) can exceed the capacity of the kidney tubules to reabsorb glucose, because the transport proteins become saturated. This result is expulsion of glucose in the urine. Glucose is a solute that draws water into the urine by osmosis. Thus, hyperglycemia causes a diabetic to produce a high volume of glucose-containing urine. In such conditions, body looses excessive amount of fluids.

The mechanism of polyuria in diabetes patients has been shown in Figure 1. The hyperglycemia results in escalated filtered load of glucose. The body strives for reabsorption of water due to loss of glucose from the body through urine.

**Fig. 2:**
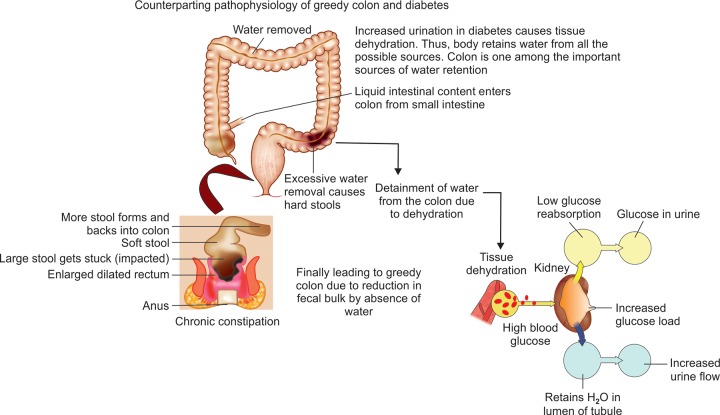
Mechanism of association of diabetes and greedy colon

Loss of the fluids and high blood glucose leads to tissue dehydration Figure 2. The body tries to absorb water from every possible source. Colon is one among these sources. The process of detainment of the water from colon due to dehydration leads to constipation or hardened stools. In this condition, the well-formed stool will be of great discomfort to the patient. The free movement of peristalsis^9^ will be disturbed by this condition. The stool in the colon will get accumulated and leads to enlarged dilated rectum.

**Fig. 3:**
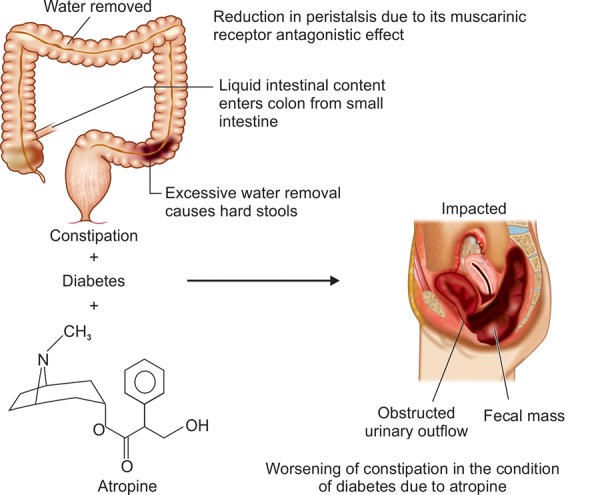
Adverseness of atropine in the condition of diabetes

### Accessible Therapy

Self-treatment of constipation with over-the-counter laxative products, home remedies and foodstuffs are common.^10^ The need to maintain good hydration is a limitation in the use of bulk-forming laxatives, in particular, in frail elderly patients. In these patients, polyethylene glycol, an osmotic agent, is an attractive alternative.^11^ Stool softeners (such as that containing docusate sodium) may be quite endorsing. Bulk laxatives, such as psyllium, may help to add fluid and bulk to the stool. Suppositories or gentle laxatives, such as milk of magnesia liquid, may help to have regular bowel movements.^12^ Enemas are to be preferred in extreme condition.^12,13^

### Atropine Usage in Diabetes Patients

Peristaltic movement or bowel movement in diabetic patients is reduced due to malfunction of the intestinal nerves. Patient feels difficulty in passing the bowel. In some worse happenings, people opt for medications like atropine in conditions, like diarrhea or motion sickness, where the excessive water loss is prevented to some extent by atropine. But at the same time, atropine shows its parasympathetic effect and leads to reduction in contractions of the stomach and intestine as shown in Figure 3. Peristalsis is incompletely suppressed and this worsens the condition in diabetes. Investigators have shown that atropine also inhibits the gastric emptying rate during hypoglycemia.^14^ Cholinergic muscarinic blockade with atropine inhibits the increase in gastric emptying during hypoglycemia. It was possible to conclude previously that the gastric emptying rates of both liquids and solid food were increased by hypoglycemia. Second, the increase in the gastric emptying rate during hypoglycemia was reversed by atropine.^14^ Atropine is a universal inhibitor of the cholinergic neurotransmitter acetylcholine and can block the effect of vagal nerve stimulation. In many cases, atropine was found to reduce the gastric emptying rate in control subjects.^14^ Evidences have shown that the mechanism of worsening of constipation in the condition of diabetes due to the muscarinic blockage effect of atropine and escalation of constipation and thus leading to more delayed or chronic constipation.

## CONCLUSION

Taken together, it is concluded that the mechanism of constipation in diabetic condition is not only due to the dysfunction in the peripheral or autonomous nervous system but also due to the agile water retention in the colon of a diabetic patient due to tissue dehydration. The detrimental side effect of atropine in the condition of diabetes is mentioned due to its muscarinic receptor antagonistic nature.

## References

[1] Shoback D, Gardner DG (2011). Greenspan’s basic and clinical endocrinology. Chapter 17..

[2] Adewole SO, Ojewole JA (2009). Protective effects ofAnnona muricata Linn (Annonaceae) leaf aqueous extract on serum lipid profiles and oxidative stress in hepatocytes of Streptozotocin-treated diabetic rats.. Afr J Trad Cam.

[3] Patel D, Kumar R, Prasad S, Sairam K (2011). Hemalathal. Antidiabetic and in vitro antioxidant potential of Hybanthus enneaspermus (Linn) F. Muell in streptozotocin-induced diabetic rats.. Asian Pac J Trop Biomed.

[4] Sarwar N, Gao P (2010). Emerging risk factors collaboration. Diabetes mellitus, fasting blood glucose concentration and risk of vascular disease: a collaborative meta-analysis of 102 prospective studies.. Lancet.

[5] Boussageon R, Bejan-Angoulvant T, Saadatian-Elahi M (2011). Effect of intensive glucose lowering treatment on all cause mortality, cardiovascular death and microvascular events in type 2 diabetes: meta-analysis of randomised controlled trials.. BMJ.

[6] Kellogg JH (1919). Form of the stool in constipation patient. Auto intoxication or intestinal toxemia. Michgan.. Modern Medicine Publishing Company of Battle Creek.

[7] Huang WS, Wang CS, Hsieh CC, Lin PY, Chin CC, Wang JY (2006). Management of patients with stercoral perforation of the sigmoid colon: report of five cases.. World J Gastroenterol.

[8] Cooke DW (2008). Plotnick. Type 1 diabetes mellitus in pediatrics.. Pediatr Rev.

[9] Ouyang A, Locke GR (2007). Overview of neurogastroenterology-gastrointestinal motility and functional GI disorders: classification, prevalence and epidemiology.. Gastroenterol Clin North Am.

[10] Bosshard W, Dreher R, Schnegg JF, Biila CJ (2004). The treatment of chronic constipation in elderly people: an update.. Drugs Aging.

[11] Camilleri M (2011). Disorders of gastrointestinal motility. Chap 138. Cecil Medicine..

[12] Lembo AJ, Ullman SP (2010). Constipation. Chap 18. Sleisenger and Fordtran’s Gastrointestinal and Liver Disease..

[13] Schvarcz E, Palmer M, Aman J, Berne C (1995). Atropine inhibits the increase in gastric emptying during hypoglycemia in humans.. Diabetes Care.

[14] Imbimbo BP, Gardino L, Palmas F, Frascio M, Canepa G, Scarpignato C (1990). Different effects of atropine and cimetropium on gastric emptying of liquids and antroduodenal motor activity in man.. Hepatogastroenterology.

